# IL-13 as Target to Reduce Cholestasis and Dysbiosis in *Abcb4* Knockout Mice

**DOI:** 10.3390/cells9091949

**Published:** 2020-08-24

**Authors:** Luisa Hahn, Nora Helmrich, Diran Herebian, Ertan Mayatepek, Uta Drebber, Eugen Domann, Stefan Olejniczak, Markus Weigel, Torsten Hain, Timo Rath, Stefan Wirtz, Hans-Joachim Mollenkopf, Nadine Schmidt, Christa Ewers, Anne Baier, Yuri Churin, Anita Windhorst, Ralf Weiskirchen, Ulrich Steinhoff, Elke Roeb, Martin Roderfeld

**Affiliations:** 1 Department of Gastroenterology, Justus-Liebig-University, D-35392 Giessen, Germany; luisahahn22@googlemail.com (L.H.); nora.helmrich@gmx.net (N.H.); Anne.S.Baier@med.uni-giessen.de (A.B.); Yury.Churin@vetmed.uni-giessen.de (Y.C.); Elke.Roeb@innere.med.uni-giessen.de (E.R.); 2 Department of General Pediatrics, Neonatology and Pediatric Cardiology, Medical Faculty, Heinrich-Heine-University, D-40225 Duesseldorf, Germany; Herebian@med.uni-duesseldorf.de (D.H.); mayatepek@med.uni-duesseldorf.de (E.M.); 3 Institute for Pathology, University Hospital of Cologne, D-50937 Cologne, Germany; uta.drebber@uk-koeln.de; 4 Institute of Medical Microbiology, German Centre for Infection Research (DZIF Partner Site Giessen-Marburg-Langen), Justus-Liebig-University, D-35392 Giessen, Germany; eugen.domann@mikrobio.med.uni-giessen.de (E.D.); Stefan.Olejniczak@dentist.med.uni-giessen.de (S.O.); Markus.Weigel@mikrobio.med.uni-giessen.de (M.W.); Torsten.Hain@mikrobio.med.uni-giessen.de (T.H.); 5 Department of Medicine 1, Division of Gastroenterology, Pneumology and Endocrinology, Friedrich-Alexander University Erlangen-Nuremberg, D-91054 Erlangen, Germany; Timo.Rath@uk-erlangen.de (T.R.); stefan.wirtz@uk-erlangen.de (S.W.); 6 Core Facility Microarray, Max Planck Institute for Infection Biology, D-10117 Berlin, Germany; mollenkopf@mpiib-berlin.mpg.de; 7 Institute of Hygiene and Infectious Diseases of Animals, Justus-Liebig-University Giessen, D-35392 Giessen, Germany; Nadine.Schmidt@vetmed.uni-giessen.de (N.S.); christa.ewers@vetmed.uni-giessen.de (C.E.); 8 Institute for Medical Informatics, Justus-Liebig-University, D-35392 Giessen, Germany; Anita.C.Windhorst@informatik.med.uni-giessen.de; 9 Institute of Molecular Pathobiochemistry, Experimental Gene Therapy and Clinical Chemistry (IFMPEGKC), University Hospital, RWTH Aachen University, D-52074 Aachen, Germany; rweiskirchen@ukaachen.de; 10 Institute for Medical Microbiology and Hospital Hygiene, Philipps University Marburg, D-35043 Marburg, Germany; ulrich.steinhoff@staff.uni-marburg.de

**Keywords:** bile acid, tight junction, Th2, liver fibrosis, bacterial translocation, intestinal microbiome

## Abstract

The Th2 cytokine IL-13 is involved in biliary epithelial injury and liver fibrosis in patients as well as in animal models. The aim of this study was to investigate IL-13 as a therapeutic target during short term and chronic intrahepatic cholestasis in an *Abcb4-*knockout mouse model (*Abcb4*^−/−^). Lack of IL-13 protected *Abcb4*^−/−^ mice transiently from cholestasis. This decrease in serum bile acids was accompanied by an enhanced excretion of bile acids and a normalization of fecal bile acid composition. In *Abcb4^−/−^/IL-13*^−/−^ double knockout mice, bacterial translocation to the liver was significantly reduced and the intestinal microbiome resembled the commensal composition in wild type animals. In addition, 52-week-old *Abcb4^−/−^IL-13*^−/−^ mice showed significantly reduced hepatic fibrosis. *Abcb4*^−/−^ mice devoid of IL-13 transiently improved cholestasis and converted the composition of the gut microbiota towards healthy conditions. This highlights IL-13 as a potential therapeutic target in biliary diseases.

## 1. Introduction

Bile ducts are involved in the formation and secretion of bile, as well as in the excretion of circulating xenobiotic substances. The intrahepatic biliary epithelium represents a barrier to diffusion of toxic substances and microbes from the bile into the interstitial hepatic tissue. Thus, the preservation of intact biliary epithelium is essential for the maintenance of its barrier function [1]. Clinically, biliary epithelial cell (BEC) barrier dysfunction is involved in the development and perpetuation of biliary diseases [2,3]. Alterations in tight junctions play a role in BEC barrier dysfunction and bile duct leakiness [4]. As a consequence, leaky intrahepatic bile ducts can cause parenchymal damage by releasing toxic bile acids (BA) and lipopolysaccharides (LPS) [2,5] into the interstitial tissue, subsequently causing inflammation and fibrosis [6].

In addition, the leaky gut hypothesis suggests that intestinal bacteria and bacterial products can translocate from the permeable gut via the portal vein into the liver. By triggering hepatic inflammation and fibrosis, a leaky gut further contributes to the progression of chronic liver disease [7]. Especially in intrahepatic cholestasis during pregnancy, a leaky gut is assumed to be involved in the hepatic pathogenesis [8]. In this context, the gut microbiota have also been claimed to be crucially involved in the pathophysiology of chronic liver diseases [9,10]. Most recently, it has been demonstrated that cholestasis changes the intestinal microbiome composition of mice, which partially resembles the observations described during human cholestasis [11].

The detergent properties of BAs are able to disrupt cell membranes and to promote the generation of reactive oxygen species. As a consequence, oxidation of lipids, proteins, and nucleic acids might cause hepatocyte necrosis and apoptosis [12].

Pro-inflammatory Th1 cytokines, such as tumor necrosis factor-alpha (TNF-α) and interferon-gamma (IFN-γ), are induced in primary sclerosing cholangitis, PSC, and biliary atresia [13,14]. These Th1 cytokines have the potential to disrupt the epithelial barrier function in cultured cholangiocytes from rodents [15,16]. In addition, Th2 cytokines, such as interleukin (IL)-4, IL-5, and IL-13, are increased in sclerosing cholangitis and play a crucial role in the desintegration of tight junction-mediated BEC barrier [17,18]. Moreover, the release of high amounts of IL-13 from type 2 innate lymphoid cells has been shown to promote hyperplasia of cholangiocytes [19]. Beyond the cytokine function in initial damage of the bile duct epithelium, a dysregulated balance of Th1 and Th2-mediated signals determines the outcome of chronic liver diseases [20]. In particular, IL-13 is able to promote hepatic fibrogenesis of different etiologies and has been identified as a major pathogenic cytokine in helminth-induced liver disease [21,22,23,24]. However, the precise functional role of IL-13 in the development of chronic cholestasis remains to be established. In this study, we carefully analyzed the impact of *IL-13* knockout on liver pathology and intestinal microbiome in *Abcb4* knockout mice.

## 2. Materials and Methods

### 2.1. Animal Model

Transgenic mice were maintained at the Central Animal Laboratory of the Justus-Liebig-University Giessen, Germany under specified pathogen-free conditions. This study was carried out in strict accordance with the recommendations in the Guide for the Care and Use of Laboratory Animals of the German law of animal welfare. Mice received humane care, and all experiments were approved by the Committee on the Ethics of Animal Experiments of the Regierungspräsidium Giessen, Giessen, Germany (permit number: V54-19c 20 15 h 01 GI20/10 No. A29/2013 and No. 128/2014). Handling and holding conditions were described previously [25]. All efforts were made to minimize suffering.

Mice were analyzed for the presence of antibodies against several mouse pathogens known to be associated with liver disease, including mouse hepatitis virus, Sendai virus, ectromelia virus, reovirus 3, and lymphocytic choriomeningitis virus, by mfd Diagnostics (Wendelsheim, Germany).

Generation and characteristics of knockout lineages C.FVB(129P2)-Abcb4^tm1Bor^ (*Abcb4*^−/−^) and BALB/c-ll13^tm2Anjm^ (*IL-13*^−/−^) have been described previously [26,27,28]. *Abcb4*^−/−^ and *IL-13*^−/−^ mice on BALB/c background were crossed to raise double-knockout hybrids C.Cg-ll13^tm2Anjm^-Abcb4^tm1Bor^ (*Abcb4*^−/−^/*IL-13*^−/−^). BALB/c WT littermates were bred from heterozygous F1 during crossbreeding to raise double-knockout hybrids. All animals in this study were bred in a single colony. At the age of 8, 26, and 52 weeks, mice were anaesthetized by isoflurane inhalation and subsequently killed by cervical dislocation. Liver samples were collected and preserved for analyses. Liver weight (LW) to body weight (BW)% ratio was calculated.

### 2.2. Routine Serum Biochemistry

Blood was collected from the vena cava. Enzymatic assays were used to measure alanine aminotransferase (ALT) as well as alkaline phosphatase (AP) on a Reflotron Plus Analyzer (Roche Diagnostics, Mannheim, Germany).

### 2.3. Histology

Sections of formalin-fixed and paraffin-embedded liver tissue were prepared and stained with hematoxylin and eosin or Sirius red. The histology of specimens was independently assessed by a pathologist specialized in hepatology (U.D.) and scored as described [29]. Briefly, portal inflammation, fibrosis, ductular reaction, mitotic activity, and Councilman bodies were scored in four grades (0–3).

### 2.4. Immunohistochemistry/Immunofluorescence

Detection of the cholangiocyte marker cytokeratin 19 (Santa Cruz Biotech., Santa Cruz, CA, USA, cat# sc-33111, RRID:AB_2234419), adhesion-marker E-Cadherin (Abcam, Berlin, Germany, cat# ab11512, RRID:AB_298118), tight-junction marker ZO-1 (Novus Biologicals, Bio-Techne GmbH, Wiesbaden, Germany, cat# NBP1-85047, RRID:AB_11023321), Ki67 (Abcam, cat# ab27619, RRID:AB_471081), SOX9 (Santa Cruz Biotech., cat# sc-20095, RRID:AB_661282), c-Jun (Cell Signaling Technology, Frankfurt am Main, Germany, cat# 9165, RRID:AB_2130165), and CD3 (Abcam, cat# ab16044, RRID:AB_443294) was performed as described [27].

### 2.5. Bile Acid Analysis

Bile acids were quantified by ultra-performance liquid chromatography-tandem mass spectrometry (UPLC-MS/MS), as has been described in the literature [30]. Bile acids from feces were extracted as described before [31].

### 2.6. Western Blot Analysis

Protein samples were prepared from total liver lysates. Protein samples were boiled in Lämmli-buffer for 5 min, chilled on ice, and subjected to 12% SDS-PAGE and transferred to polyvinylidene difluoride membranes. Visualization of proteins was performed by horseradish-peroxidase (HRP)-linked antibodies. ECL Chemiluminescence Detection Kit (SuperSignal West Pico Chemiluminescent Substrate, Thermo Scientific, Darmstadt, Germany) was used according to the manufacturer’s protocol.

### 2.7. Transcriptome Microarray and Gene Set Analysis

Microarray analysis was performed with total RNA from the liver of 8-week-old mice as described previously [32]. The data presented here have been deposited in NCBI’s Gene Expression Omnibus (GEO; http://www.ncbi.nlm.nih.gov/geo/, RRID:SCR_004584) and are accessible through GEO Series accession number GSE90995. *p*-values less than 0.01 and twofold changes were defined as the cut-off criteria. Functional annotation clustering of the large lists of genes that were regulated was performed using the Database for Annotation, Visualization and Integrated Discovery at default settings (DAVID 6.8, RRID:SCR_001881) [33] and Kyoto Encyclopedia of Genes and Genomes (KEGG, RRID:SCR_012773) [34].

### 2.8. Bacterial Translocation to the Liver

Homogenates of 50mg liver tissue were filtered using a cell strainer (100 μm pore size; Corning, Tewksbury, MA 01876 USA) and serial dilutions were plated onto a blood agar plate (Thermo Scientific^™^ Oxoid^™^, Fisher Scientific, Wesel, Germany), brain heart infusion (BHI) agar (Thermo Scientific™), and water-blue metachrome-yellow lactose agar (acc. to Merck KGaA, Darmstadt, Germany). Additionally, Schaedler agar (Becton Dickinson GmbH, Heidelberg, Germany) was used for anaerobic growth examination in an anaerobic jar system (AnaeroGen^™^; Thermo Scientific^™^ Oxoid^™^, Fisher Scientific, Oxford, OX14 4SD, UK) after 72 h of incubation at 37 °C. For enrichment purposes, each sample was inoculated to nutrient broth no. 2 containing 10% bovine serum and broth were streaked onto blood, BHI and Schaedler agar plate after 24 h incubation at 37 °C. Plates were examined after 24 and 48 h, as well as for BHI and Schaedler agar after 72 h at 10% CO_2_. Morphologically diverse colonies were sub-cultivated. Pure cultures were identified using MALDI-TOF MS (Biotyper Version V3.3.1.0, Bruker Daltonics, Bremen, Germany) by the direct smear method and information provided in the DB7311 database. The MALDI-TOF results were supported by the Gram stain method and morphological properties of the bacteria.

### 2.9. Quantitative Real-Time PCR

RNA isolation, complementary DNA synthesis and real-time PCR were performed as described previously [35]. Oligonucleotide sequences are available upon request. We used 18S ribosomal RNA as housekeeping control.

### 2.10. Hepatic Hydroxyproline Ccontent

To quantify liver fibrosis, hepatic hydroxyproline was measured as described previously [36].

### 2.11. 16 SrDNA Sequencing forMicrobiome Profiling

Mouse feces was collected in sterile 1.5 mL Eppendorf tubes, weighted, and filled-up with 0.9% NaCl in order to adjust a concentration of 0.1 g/mL. The samples were extensively vortexed and DNA was extracted by using glass beads and the Power Lyzer DNA Isolation Kit from MoBio as recommended by the vendor (MoBio Laboratories, Carlsbad, CA, USA). DNA was eluted with 100 µL of DNase-free water and concentration was determined using Qubit Fluorometric Quantitation (Thermo Fisher Scientific, Waltham, MA, USA). The V4 region of 16S rRNA gene was amplified using adapter forward primer 5′-TCG TCG GCA GCG TCA GAT GTG TAT AAG AGA CAG GTG CCA GCM GCC GCG GTA A-3′, adapter reverse primer 5′-GTC TCG TGG GCT CGG AGA TGT GTA TAA GAG ACA GGG ACT ACH VGG GTW TCT AAT-3′ and the 2 × Kapa HiFi HotStart Ready Mix (Kapa Biosystems, Wilmington, MA, USA). The amplification profile comprised an initial heating step at 95 °C for 30 s, 25 cycles of denaturation at 95 °C for 30 s, annealing at 55 °C for 30 s, elongation at 72 °C for 30 s, and a final elongation step at 72 °C for 5 min. PCR products were purified with Agencourt AMPure XP system as recommended by the vendor (Beckman Coulter, Brea, CA, USA). The size, purity, and concentration of amplicons were determined using the Agilent Bioanalyzer as recommended by the vendor (Agilent Technologies, Santa Clara, CA, USA). The index PCR was done using the Nextera index Kit v2 Set B as recommended by the vendor (Illumina, San Diego, CA, USA). The quality of the index PCR was determined as described above for the adapter PCR. The library was adjusted to 3 pM, the flow cell was prepared and loaded according to the Reagent Preparation Guide of MiSeq Reagent Kit v2 as recommended by the vendor (Illumina) and sequenced using Illumina’s MiSeq device.

### 2.12. Bioinformatics Workflow

MiSeq software version 2.6 was used to split the sequences by barcode and to generate the fastq files. The microbiome analysis was done following the MiSeq Soap [37] using Mothur version 1.36.1 [37]. For the alpha and beta diversity calculation and the taxa summary plots, Qiime version 1.9.1 [38] was used. The paired end reads were joined, and the primer sequences were removed. We filtered for the expected amplicon length and removed reads with ambiguous base calls or with homopolymers longer the eighth nucleotides. Duplicate sequences were merged. The unique reads were aligned against the SILVA-bases bacterial reference alignment [39]. Nucleotides outside the expected alignment region were trimmed. Reads with a difference of two nucleotides were merged during pre-clustering. Chimeric reads were removed using the Mothur implementation of the UCHIME algorithm [40]. After chimera removal, the taxonomy was assigned and non-bacterial reads were discarded. The Operational taxonomic units (OTUs) were created using the cluster split method of Mothur. After clustering, we reassigned the taxonomy to the OTUs. In preparation for the analysis with Qiime, a phylogenetic tree and an OTU table in biom format were created. Alpha and beta diversity analysis and the taxa summery plots were created using the Qiime core diversity analysis script.

### 2.13. Statistical Analysis

Statistical analysis was performed with SPSS V. 22.0 software (SPSS Inc.). The significance of the data was determined by Kruskal–Wallis tests and subsequent Mann–Whitney U tests as well as bivariate Spearman’s rank tests and, the results were considered significant at *p* < 0.05. Sparse partial least squares discriminant analysis (sPLSDA) was conducted with R (Version 3.1.1) and the mixOmics R-package (version 6.0.0). The method sPLSDA is based on classical partial least squares regression and was used for classification and discrimination problems. In addition, sPLSDA allows variable selection. Here, we used sPLSDA to discriminate between the different mouse lines by their microbiome. For network analysis, the strongest relationships between strains from the microbiome and mouse strains are depicted. The threshold is set at an absolute association value of 0.51. The heatmap gives a representation of the similarity matrix between mouse lines, as well as between strains of the microbiome. A hierarchical clustering is applied to mouse lines, as well as to strains of the microbiome. The results of the hierarchical clustering are depicted in dendrograms at the sides of the heatmap.

## 3. Results

### 3.1. IL-13 Knockout Restored Intrahepatic Bile Duct Integrity and Biliary Epithelial Cell Barrier Function in Abcb4^−/−^ Mice

**Figure 1:**
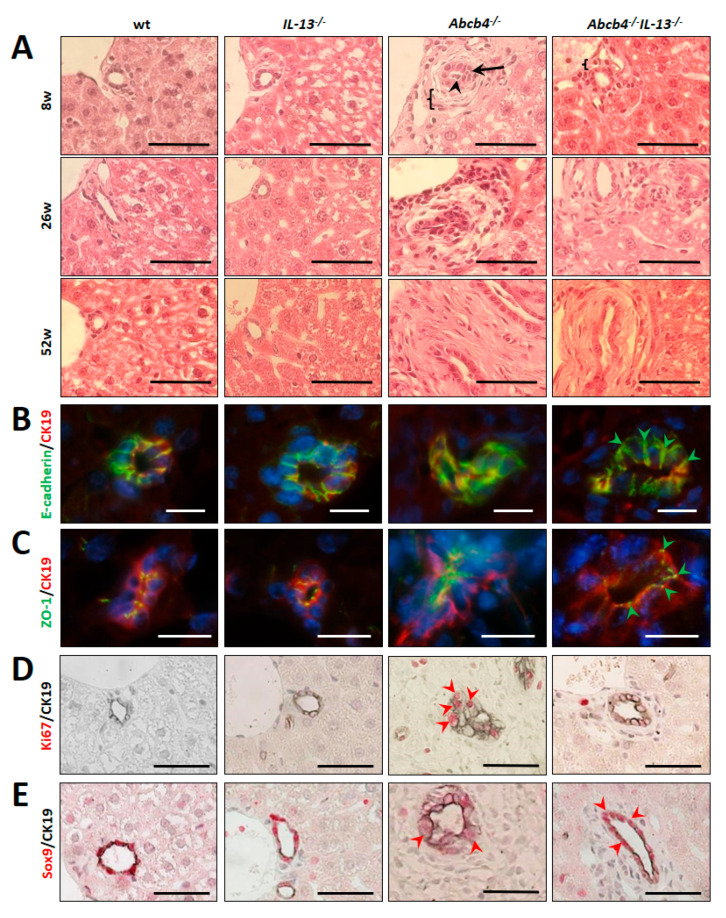
*IL-13* knockout restored bile duct architecture in *Abcb4*^−/−^ mice. (**A**) Hematoxylin and eosin (H & E)-staining demonstrated loss of lumenization or bile duct collapse (arrowhead), massive proliferation of biliary cells (arrow), thickening of mesenchymal layer (bracket), swelling of biliary epithelial cells (BECs), and altered BEC organization (arrows) in *Abcb4*^−/−^ mice. In contrast, appearance of BEC and bile duct morphology was preserved in *Abcb4*^−/−^ mice with *IL-13* knockout and biliary architecture were preserved (arrowheads). H & E staining and histological scoring by an expert (U.D.) were performed for all mice in this study (*n* = 10 per group, 120 sections). (**B**,**C**) Immunostaining of CK19 (red) and (**B**) E-cadherin (green) as well as (**C**) ZO-1 (green) accentuated structural preservation of distinct biliary cell contacts and sharply stained tight junctions in *Abcb4^−/−^/IL-13*^−/−^ mice in comparison to *Abcb4*^−/−^ mice (arrowheads). *Abcb4*^−/−^ showed altered tight junction morphology characterized by an altered, partially disrupted, ZO-1 staining pattern between BECs. (**D**) Ki67 staining (red) indicated proliferation of CK19^+^-BEC in *Abcb4*^−/−^ mice (arrowheads). (**E**) Homogenous staining for BEC-marker Sox9 (red) is almost lost in CK19^+^ (black) BEC for *Abcb4*^−/−^ mice but largely preserved in *Abcb4^−/−^/IL-13*^−/−^ mice (arrowheads). Immunohistochemistry was performed for 3-4 representative animals per group in 8-week-old animals, 12–16 sections per immunostaining). Representative micrographs are shown. Bars (**A**,**D**,**E**) 50 µm and (**B**,**C**) 12 µm, magnification (**A**,**D**,**E**) ×400 and (**B**,**C**) ×1000.

Livers of *Abcb4*^−/−^ mice showed distorted bile canaliculi and areas of dilatation, while the morphology of BEC and the bile ducts was preserved in *Abcb4*^−/−^*/IL-13*^−/−^ mice (Figure 1A). In detail, loss of lumenization or bile duct collapse (arrowhead), proliferation of biliary cells (arrows), thickening of the mesenchymal layer (brackets), swelling of BEC, and altered BEC organization were ameliorated in *Abcb4*^−/−^*/IL-13*^−/−^ *mice* (Figure 1A). However, elderly *Abcb4*^−/−^*/IL-13*^−/−^ mice (52 weeks) showed the same pattern of perturbed intrahepatic bile duct architecture as *Abcb4*^−/−^ *mice* (lower right panels of Figure 1A). Since loss of epithelial barrier function has been identified to cause regurgitation of bile acids from leaky bile ducts in *Abcb4*^−/−^ mice [4], we analyzed the expression of tight junctions via immunofluorescence staining of E-cadherin and ZO-1. Cell–cell contacts between BECs were intact in 8-week-old *Abcb4*^−/−^*/IL-13*^−/−^ mice, but not in age-matched *Abcb4*^−/−^, illustrating that barrier function is preserved upon the genetic deletion of *IL-13* in *Abcb4*^−/−^ mice (Figure 1B,C). In order to determine proliferation and differentiation of BECs, Ki67 and SRY-related HMG box transcription factor 9 (Sox9) were immunohistochemically analyzed in combination with the BEC-marker, CK19 (Figure 1D,E).

While proliferation was a rare event in BECs of controls and in *Abcb4*^−/−^*/IL-13*^−/−^ mice, approx. 50% of BECs stained positive for Ki67 in Abcb4^−/−^ mice, indicating that *IL-13* knockout reduced biliary proliferation in *Abcb4*^−/−^ mice (Figure 1D and Figure S1). Biliary Sox9 was only marginally expressed in *Abcb4*^−/−^ mice while BECs of controls and *Abcb4^−/−^/IL-13*^−/−^ mice strongly expressed Sox9, which is important for bile duct morphogenesis and cholangiocyte polarity [41] (Figure 1E). Regurgitation of bile acids from leaky bile ducts is the pathological key event leading to enhanced hepatic- and serum BA concentrations that finally cause hepatobiliary disease in *Abcb4*^−/−^ mice [4].

**Figure 2:**
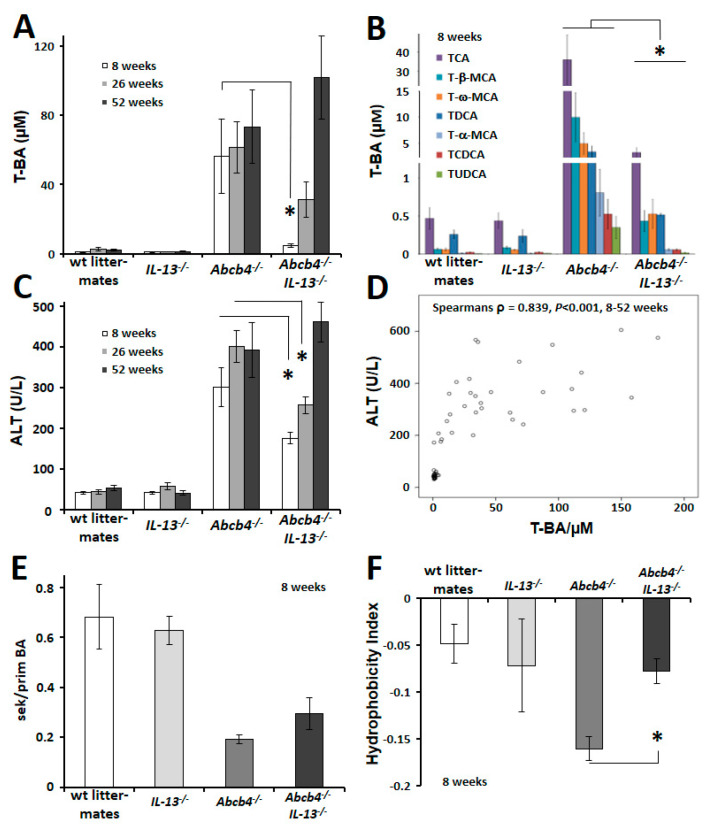
*IL-13*-knockout reduced serum bile acid concentrations and improved hepatic integrity. (**A**) IL-13-knockout transiently reduced serum bile acids in *Abcb4^−/−^/IL-13*^−/−^ mice. (**B**) The seven most prominent serum bile acids were significantly reduced in 8-week-old *Abcb4^−/−^/IL-13*^−/−^ mice. Wt and *IL-13*^−/−^ controls *n* = 3–4, *Abcb4*^−/−^ and *Abcb4^−/−^/IL-13^−/−^ n* = 4–6. Please note the two scale breaks in the *y*-axis. (**C**) Serum biochemical parameter of hepatocellular injury (ALT) decreased in *Abcb4^−/−^/IL-13*^−/−^ mice of 8 and 26 weeks but adjusted to *Abcb4*^−/−^-serum levels at 52 weeks of age. *n* = 8–12. (**D**) Serum ALT correlated well to serum bile acid concentrations (8, 26, and 52 weeks). (**E**) The ratio between secondary and primary serum bile acids was not altered by IL-13 knockout in *Abcb4*^−/−^ mice at the age of 8 weeks. (**F**) The hydrophobicity index was normalized in *Abcb4^−/−^/IL-13*^−/−^ mice aged 8 weeks. Values are means ± SEM. * *p* < 0.05 Abcb4^−/−^ vs *Abcb4^−/−^/IL-13*^−/−^. Abbreviations used are: T-BA, taurine conjugated bile acids; wt, wild type.

Consistent with the preserved barrier function in *Abcb4^−/−^/IL-13*^−/−^ mice, quantitative analysis of serum bile acids in these mice revealed a ten-fold decrease compared to *Abcb4*^−/−^ (*p* = 0.014, Figure 2A, significances between *Abcb4*^−/−^- and *Abcb4^−/−^/IL-13*^−/−^-groups are highlighted). Furthermore, analysis of the seven most prominent BAs revealed a significant decline in each BA in double knockout mice compared to *Abcb4*^−/−^ mice (Figure 2B). Unconjugated and glycine-conjugated BAs represented less than 5% of the total serum-bile acid pool in both, *Abcb4*^−/−^ and *Abcb4^−/−^/IL-13*^−/−^ mice (not shown).

### 3.2. Reduced Serum BA Improved Liver Integrity

Although *Abcb4^−/−^/IL-13*^−/−^ mice exhibit the same phenotype with respect to appearance, fertility, survival, body and liver weight as *Abcb4*^−/−^ controls, we observed 1.7- and 1.6-fold reduced serum ALT levels in 8- and 26-week-old *Abcb4^−/−^/IL-13*^−/−^ mice, respectively (Figure 2C). As serum ALT correlated with serum BA concentrations, we suggest that biliary destruction precedes chronic liver injury (ρ = 0.839, *p* < 0.001, Figure 2D). In order to define the impact of IL-13 on bile acid composition, the ratio of secondary to primary BAs and the hydrophobicity index were calculated by a modified Heuman’s method (Figure 2E,F) [42,43]. Surprisingly, the characteristic ratio of secondary to primary BA, which is specific for liver damage, was not altered in 8-week-old *IL-13* deficient *Abcb4*^−/−^ mice (Figure 2E). However, the hydrophobicity index (HI) of serum BA was reduced in 8-week-old *Abcb4*^−/−^ mice as compared to wild type controls, indicating increased hydrophilic BAs (*p* = 0.014, Figure 2F). Most importantly, HI became normal in *Abcb4^−/−^/IL-13*^−/−^ animals. Neither alkaline phosphatase (Figure S2) nor the expression of genes responsible for BA synthesis and transport, as well as hepatic inflammation, were altered in *Abcb4^−/−^/IL-13*^−/−^ mice, as compared to *Abcb4*^−/−^-controls (Figure S3). Interestingly, ER-stress-associated PERK and eIF2α were phosphorylated in *Abcb4*^−/−^ mice, while this phosphorylation pattern was reduced in *Abcb4^−/−^/IL-13*^−/−^ mice (Figure S4).

### 3.3. Lack of IL-13 Reduces Hepatic Pathology and Fibrosis

**Figure 3:**
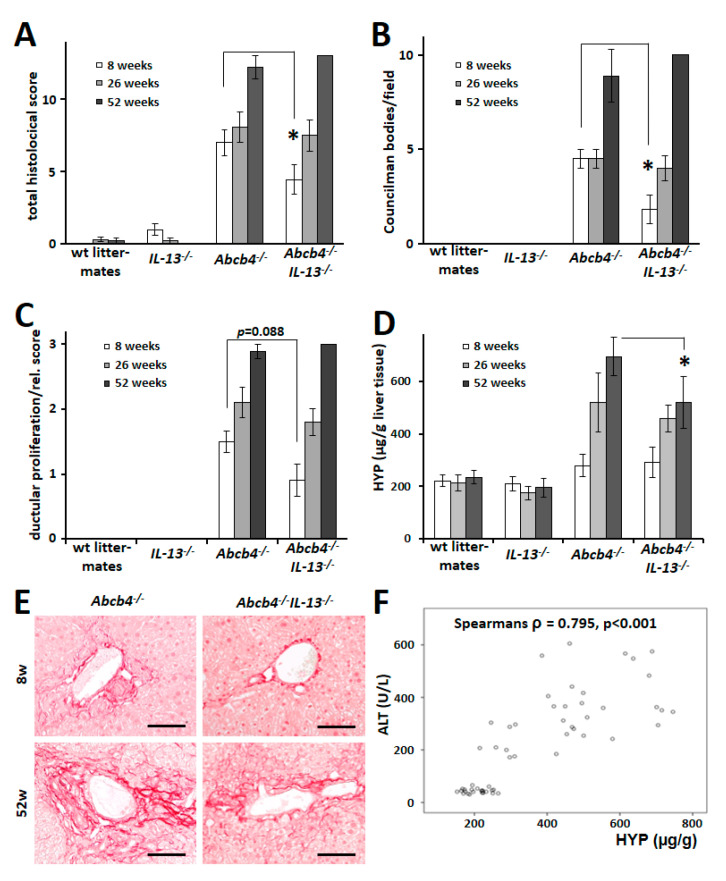
*IL13* knockout reduced hepatic pathology and fibrosis in *Abcb4*^−/−^ mice. (**A**–**C**) Histological scoring revealed an ameliorated total histological score (**A**) and a reduced number of apoptotic hepatocytes (**B**) in 8-week-old *Abcb4^−/−^/IL-13*^−/−^ mice (*p* = 0.047 and *p* = 0.018, respectively), while pathologic assessment of bile duct proliferation failed to reach statistical difference (**C**). (**D**) Quantitative assessment of hydroxyproline (HYP) showed reduced hepatic accumulation of fibrillary collagen in 52-week-old *Abcb4^−/−^/IL-13*^−/−^ mice, Values are means ± SEM. * *p* < 0.05 Abcb4^−/−^ vs *Abcb4^−/−^/IL-13*^−/−^. (**E**) Sirius-red staining demonstrated reduced chicken wire fibrosis along with sinusoids and loosened periportal fibrillar collagen deposition in 52-week-old *Abcb4^−/−^/IL-13*^−/−^ mice. Magnification 200 ×, bars 100 µm. (**F**) Serum ALT correlated well with hepatic HYP content.

IL-13-devoid *Abcb4*^−/−^ mice had a reduced histological score with decreased numbers of apoptotic hepatocytes in 8-week-old animals (Figure 3A,B). Despite reduced expression of the proliferation marker Ki67 in *Abcb4^−/−^/IL-13*^−/−^ mice, histological assessment of ductular proliferation did not show significant changes (Figure 3C). Hepatic fibrosis was clearly reduced in 52-week-old *Abcb4^−/−^/IL-13*^−/−^ mice (*p* = 0.002, Figure 3D,E). ALT was highly correlated with HYP as a marker of fibrogenesis (Figure 3F).

### 3.4. IL-13 Alters Hepatic Gene Expression

In order to investigate the impact of IL-13 on gene regulation, we performed an RNA-array from the livers of *Abcb4*^−/−^ mice and *Abcb4^−/−^/IL-13*^−/−^ mice. IL-13 depletion caused changes in the expression pattern of genes as compared to the livers of IL-13-proficient *Abcb4*^−/−^ mice. Numerous transcripts were regulated more than two-fold with *p* < 0.05 in *Abcb4*^−/−^ mice as compared to *Abcb4^−/−^IL-13*^−/−^ mice (749 vs 569, respectively). Functional annotation clustering revealed biological processes that are crucially involved in the development of cholestasis in *Abcb4^−/−^ mice* (Table 1). For example, genes related to cell adhesion and ECM receptor interaction are likely to be involved in the modulation of BEC barrier function, while genes related to the extracellular matrix are linked to fibrogenesis.

Of note, UDP glucuronosyltransferase 2 B37 (*Ugt2b37*) and *Cyp26* encoding for enzymes involved in retinoic acid and estrogen clearance were strongly upregulated in *Abcb4*^−/−^ and suppressed in *Abcb4^−/−^/IL-13*^−/−^ mice. On the other hand, genes involved in metabolite biosynthesis and transport such as *Cyp3a41b* and *Cyp3a44* were strongly reduced in *Abcb4*^−/−^ and induced in *Abcb4^−/−^/IL-13*^−/−^ mice. A similar regulation was observed for genes related to the PPAR signaling pathway, including members of the *Cyp4a12* family (involved in blood pressure) but also fatty acid binding proteins, involved in fatty acid transport and lipid accumulation in the liver. Thus, global gene analysis identified many genes that directly or indirectly are involved in IL-13-regulated biliary pathology. Nevertheless, array analysis and functional annotation clustering did not demonstrate different regulation of BA transporters and pumps between *Abcb4*^−/−^ and *Abcb4^−/−^/IL-13*^−/−^ mice. The data presented here have been deposited in NCBI’s Gene Expression Omnibus (GEO; http://www.ncbi.nlm.nih.gov/geo/, RRID:SCR_004584) and are accessible through GEO Series accession number GSE90995.

### 3.5. Lack of IL-13 Alters Bile Acid Excretion Patterns, Improves Ileal Integrity, and Reduces Bacterial Translocation

**Figure 4:**
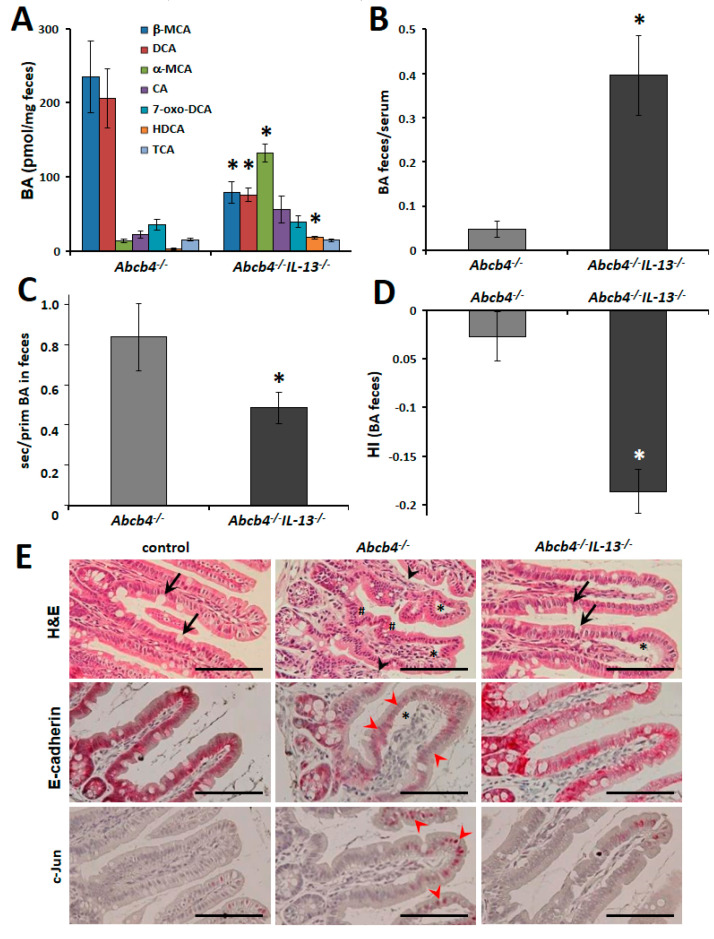
Restored bile acid excretion pattern improved ileal integrity. (**A**) Two of the seven most concentrated bile acids were significantly reduced in the feces of 8-week-old *Abcb4^−/−^/IL-13*^−/−^ mice, while the concentration of three bile acids were increased. (**B**) The ratio of bile acid excretion (feces/serum) was increased 8-fold in 8-week-old *Abcb4^−/−^/IL-13*^−/−^ mice. (**C,D**) The ratios of secondary to primary bile acids (**C**) and hydrophobicity index (**D**) of fecal bile acids were reduced in feces of 8-week-old *Abcb4^−/−^/IL-13*^−/−^ mice. Values are means ± SEM for *n* = 5 (*Abcb4*^−/−^) and *n* = 6 (*Abcb4^−/−^/IL-13*^−/−^) for fecal BA analysis. * *p* < 0.05 *Abcb4*^−/−^ vs. *Abcb4^−/−^/IL-13*^−/−^. BA bile acid, wt wild type. (**E**) H&E staining as well as immunostaining for E-cadherin and c-Jun demonstrate blunted and crippled villi (#), loss of goblet cells (arrows), and separation of villus epithelium (*) from underlying lamina propria in *Abcb4*^−/−^ mice and amelioration of these symptoms in *Abcb4^−/−^/IL-13^−/−^ mice*. Note the tremendous loss of E-cadherin and the distinct nuclear c-Jun-staining in epithelia at the tips of villi in *Abcb4*^−/−^ mice (red arrowheads, middle and lower panels). Magnification 200×, bars 100 µm. Abbreviation: HI hydrophobicity index.

The observation of strongly reduced serum BA concentrations but unaltered BA synthesis and BA transport raised the question if the composition of excreted bile acids was also modified in *Abcb4^−/−^/IL-13*^−/−^ mice. Quantitative analysis of BAs in feces revealed an entirely modified composition of excreted BAs (Figure 4A). While β-muricholate (β-MCA, *p* = 0.018) and deoxycholate (DCA, *p* = 0.018) are the major BAs excreted by *Abcb4*^−/−^ mice, we observed that the diversity of excreted BAs was by far bigger in *Abcb4^−/−^/IL-13*^−/−^ mice (Figure 4A). α-MCA (*p* = 0.006) and β-MCA were predominantly found in the feces of *Abcb4^−/−^/IL-13*^−/−^ mice. Interestingly, hydrophilic Hyodeoxycholic acid (HDCA) was one of the major BAs excreted by double knockout mice (*p* = 0.006, Figure 4A).

**Figure 5:**
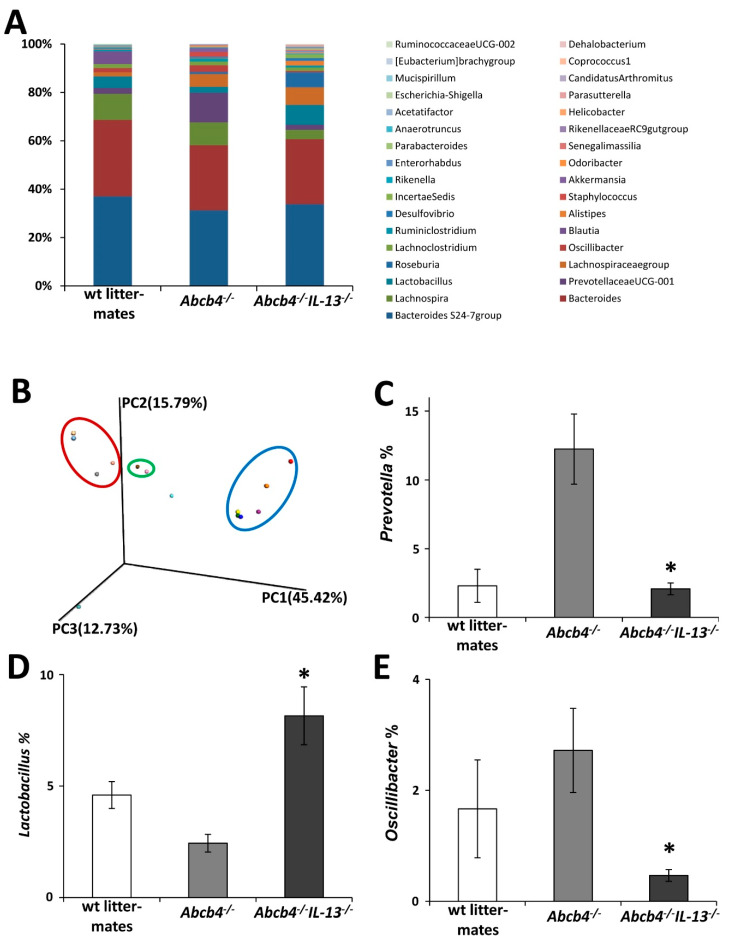
Beneficial change in intestinal microbiome in *Abcb4^−/−^/IL-13*^−/−^ mice. Next-generation sequencing demonstrates alterations of the intestinal microbiome (**A**), while subsequent principle component analysis revealed characteristic and group specific compositions (**B**). Deleterious bacteria such as *Prevotellaceae* (**C**) and *Oscillobacter* (**E**) were raised in *Abcb4*^−/−^ mice and normalized by the additional *IL-13*^−/−^, while beneficial microbiota such as *Lactobacillae* (**D**) were raised in *Abcb4^−/−^/IL-13*^−/−^ mice in comparison to *Abcb4*^−/−^. Values are means ± SEM. * *p* < 0.05 Abcb4^−/−^ vs *Abcb4^−/−^/IL-13*^−/−^.

The ratio of excreted serum BAs was increased eight-fold (*p* = 0.006) in *Abcb4^−/−^/IL-13*^−/−^ mice, while the total amount of BAs excretion was not altered by IL-13 depletion in *Abcb4*^−/−^ mice (Figure 4B). The ratio of secondary to primary BAs and hydrophobicity index were reduced in *Abcb4^−/−^/IL-13*^−/−^ mice (*p* = 0.018, Figure 4C,D). With the knowledge that BAs with increased hydrophobicity display enhanced toxicity [44,45], we next studied the ileal histopathology of IL-13-proficient and -deficient *Abcb4*^−/−^ mice (Figure 4E and Figure 5). In *Abcb4*^−/−^ mice, blunting and crippledness of villi as well as loss of goblet cells indicated intestinal pathology (Figure 4E). Separation of the villus epithelium from the underlying lamina propria indicated pronounced interstitial edema (stars, Figure 4E). These pathological alterations were less pronounced in *Abcb4^−/−^/IL-13*^−/−^ mice (upper right panel Figure 4E). Similarly, inconsistent and reduced expression of E-cadherin as well as enhanced c-Jun expression was normalized in *Abcb4^−/−^/IL-13^−/−^ mice* (middle and lower panels, Figure 4E). Furthermore, epithelial proliferation and lymphocyte infiltration were close to normal in *Abcb4^−/−^/IL-13*^−/−^ mice (Figure S5). An overview of the histological alterations is shown in Figure S5C–E. Furthermore, we analyzed bacterial translocation to the liver, which has also been observed in other models of cholestasis [46]. Bacterial translocation to the liver was reduced in *Abcb4^−/−^/IL-13*^−/−^-mice. (Figure S6).

### 3.6. IL-13 Depletion is Associated with Profound Compositional Changes in Gut Microbiota

**Figure 6:**
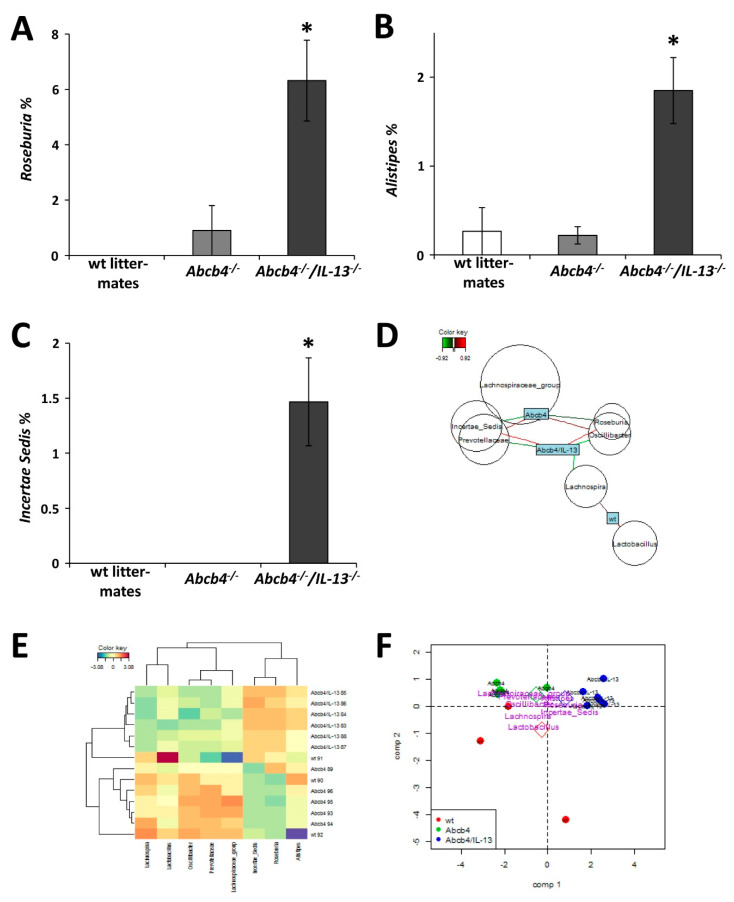
*IL-13* knockout reversed specific intestinal microbiome associations in cholestatic mice. (**A**–**C**) Intestinal occurrence of *Roseburia*, *Alistipes*, and *Incertae Sedis* was increased in *Abcb4^−/−^/IL-13*^−/−^ mice. Mean ± SEM is depicted, * *p* < 0.05 was considered significant. (**D**) Network displays the negative association between *Abcb4*^−/−^ mice and *Roseburia*, and *Incertae Sedis* (green edges) and the positive association (red edges) with *Prevotella*, and *Oscillibacter*. In *Abcb4^−/−^/IL-13*^−/−^ mice, these associations are in reverse. In addition, *Abcb4^−/−^/IL-13*^−/−^ mice are negatively associated with *Lachnospira*. (**E**) Heatmap and hierarchical clustering of different mice indicates a high similarity between *Abcb4*^−/−^ and wildtype mice, due to the decreased occurrence of *Roseburia*, *Alistipes*, and *Incertae Sedis* in *Abcb4*^−/−^ mice and the increased occurrence of *Prevotella*, the group of *Lachnospiraceae*, and *Oscillibacter*. (**F**) The biplot clearly shows a distinction between *Abcb4^−/−^/IL-13*^−/−^ mice and *Abcb4*^−/−^ mice.

Altered BA composition in feces and ileal integrity raised the question whether the intestinal microbiome was also affected in IL-13 deficient mice. 16S RNA sequencing revealed that IL-13 impacted the composition of the intestinal microbiome in *Abcb4*^−/−^ mice (Figure 5A). Sparse partial least-square discriminant analysis revealed characteristic and group specific composition of fecal microbiomes (Figure 5B). For example, bacteria belonging to the families of *Prevotellaceae* and *Oscillibacter* were overrepresented in *Abcb4*^−/−^ mice and normalized in *Abcb4^−/−^/IL-13* knockout, while *Lactobacillae*, *Roseburia*, *Alistipes*, and *Incertae Sedis* were increased in *Abcb4^−/−^/IL-13*^−/−^ mice (Figure 5C–E and Figure 6).

## 4. Discussion

Impaired bile formation or flow is the precondition for cholestatic liver diseases which can lead to chronic liver injury and end-stage liver disease. It has been demonstrated that the regurgitation of bile acids from leaky bile ducts causes sclerosing cholangitis [4]. The Th2-specific cytokine IL-13 is involved in the disruption of the BEC barrier in patients with IgG4-related cholangitis [17] and promoted cholangiocyte hyperplasia [19]. On the other hand, a Th1-shift improved cholestasis in *Abcb4*^−/−^ mice [27]. In the present study, we analyzed the effect of IL-13 on cholestasis and on the intestinal microbiome in *Abcb4*^−/−^ mice and found that the absence of IL-13 improved cholestasis and hepatic fibrosis in *Abcb4*^−/−^ mice. Further, IL-13 deficient *Abcb4*^−/−^ mice revealed normal composition of bile acids, restored intestinal integrity, and preservation of commensal intestinal microbiota.

*Abcb4*^−/−^ mice represent a well-characterized model for chronic cholestatic liver diseases [4]. Biliary injury and subsequent progressive injury of hepatic parenchyma result from defective biliary phospholipid (PL) secretion and subsequent increase in free non-micellar and toxic BA concentrations [4]. Loss of epithelial barrier function in *Abcb4*^−/−^ mice is due to disturbed expression of tight junctions, altered basement membranes, and abnormally widened intercellular spaces of BECs [4]. Our data show profound alterations of intercellular BEC junctions in *Abcb4*^−/−^ mice, indicated by altered expression pattern of E-cadherin and zonula occludens-1 (ZO-1). E-cadherin and ZO-1 play an important role in the maintenance of epithelial cell polarity and exhibit selective permeability to molecules with respect to their charge and size [1,47,48,49]. The loss of intercellular BEC junctions in *Abcb4*^−/−^ mice was almost normalized in *Abcb4^−/−^/IL-13*^−/−^ mice, which is in line with studies showing the disruption of the tight junction-associated BEC barrier by Th2 signals in cholangitis and biliary atresia [17,50]. Additionally, IL-13 reduced tight junction-associated BEC barrier function [17] as well as epithelial hyperplasia and biliary repair in vitro [19]. In agreement with these findings, we found that proliferation of BEC was reduced in *Abcb4^−/−^/IL-13*^−/−^ animals as compared to *Abcb4*^−/−^-controls. Furthermore, while the biliary protein Sox9 was barely detectable in *Abcb4*^−/−^ mice, normal expression was seen in *Abcb4^−/−^/IL-13*^−/−^ mice. Lack of biliary proliferation and sustained Sox9 expression, both of which are required for bile duct morphogenesis and cholangiocyte polarity [41], demonstrates that IL-13 is essentially involved in the ductular reaction of *Abcb4*^−/−^ mice. Functional clustering of regulated genes further supports the role of IL-13 to interfere with the integrity of ductular cells. In *Abcb4^−/−^/IL-13*^−/−^ mice, the protective effect was associated with a 10-fold reduction in toxic serum-bile acid concentrations [51]. Thus, reduced serum ALT and improved pathological scoring clearly demonstrate preserved hepatic integrity.

Interestingly, the beneficial effects of IL-13 are obviously transient. Thus, the progression of liver disease could not be prevented but delayed through prolonged preservation of the biliary epithelium. This observation underlines that epithelial barrier function is crucial for the initiation of hepatic damage in *Abcb4*^−/−^; however, the loss of biliary phosphatidylcholine was not compensated by the lack of IL-13.

Recently, it has been demonstrated that IL-13 simultaneously and independently promoted hepatic fibrosis and the biliary reaction to progressive liver disease [52]. The clinical impact of IL-13 and downstream signaling events in chronic liver diseases of different etiologies and fibrosis were recently summarized [22]. Both fibroblasts and biliary cells are directly targeted by IL-13, resulting in the activation of ECM-producing myofibroblasts and concurrent ductular reaction [52]. Therefore, it is likely that reduced fibrosis in 52-week-old *Abcb4^−/−^/IL-13*^−/−^ mice is a cumulative effect of delayed parenchymal injury caused by preserved BEC barrier function and decreased activation of ECM-producing myofibroblasts.

Bile acid-induced endoplasmic reticulum stress has been shown to stimulate apoptosis in HepG2 cells in a hydrophobicity- and concentration-dependent manner [53]. In our model, the impact of ER-stress on hepatocyte damage during cholestasis is shown by the phosphorylation of ER-stress-dependent PERK and eIF2α in *Abcb4*^−/−^ mice, which was reduced by the *IL-13*^−/−^-knockout.

In the current study, the global transcript profile of liver tissue revealed that gene expression in *Abcb4*^−/−^ mice significantly differed from wild type and was almost comparable to controls in *Abcb4^−/−^/IL-13*^−/−^ mice: (i) reduced expression of intercellular adhesion molecules in the presence of IL-13 supports the idea that depletion of this cytokine has stabilizing effects on BEC barrier function [54]. (ii) Steroid hormone metabolism was significantly altered in Abcb4^−/−^ mice in comparison to wild type mice and returned to normal in *Abcb4^−/−^/IL-13*^−/−^ mice. Furthermore, functional annotation clustering of regulated genes indicated alterations in biological processes that may be involved in ductular integrity and fibrosis. Previous studies demonstrated that retinol and steroid metabolism as well as PPAR signaling pathway might be linked to liver damage in *Abcb4*^−/−^ mice. Modulation of PPAR- and retinoic acid signaling in clinical and animal studies showed promising results for the therapy of cholangiopathies [55]. Modulation of PPAR alters hyperlipidemia, bile acid metabolism, inflammation, detoxification, and fibrosis [29,55], while all-trans retinoic acids have been shown to regulate cell proliferation, differentiation, and morphogenesis, as well as inflammation in biliary diseases [55,56]. Our study revealed that biochemical conversion of all-trans retinoic acids was differentially regulated in *Abcb4*^−/−^ as compared to *Abcb4^−/−^/IL-13*^−/−^ mice.

It has been assumed that IL-13 is a key effector cytokine in ulcerative colitis and that enterohepatic bile acid circulation is disturbed by BA malabsorption in inflammatory bowel diseases [57,58,59]. Increased luminal bile acid hydrophobicity was associated with cytotoxicity and contributes to gut barrier dysfunction [60], which is also reflected by our current study. Furthermore, in alcohol-induced injury of the gut barrier, augmented secondary BAs were associated with enhanced inflammation of the colonic mucosa [61]. In contrast, IL-13 deficiency caused reduction in secondary fecal bile acids in *Abcb4*^−/−^ mice, resulting in marginal ileal damage. Although the inflammatory functions of BA have been known for a long time, they are not primary initiators of the pathologies [62]. We assume that the enhanced pathology seen in *Abcb4*^−/−^ mice is due to mutual interference of hepatic and ileal damage, underlining the enterohepatic relationship in liver injury. Thus, normalization of BA excretion and sustained ileal integrity in *Abcb4^−/−^/IL-13*^−/−^ mice preserved the circulation of enterohepatic BA.

Bacterial infections are frequent in cirrhosis and contribute to the progression of chronic liver disease [7]. It has been suggested that cholestasis-associated bacterial translocation may be linked to enhanced levels of intestinal cytokines such as IFN-γ, IL-4, and IL-17 that facilitate the intestinal permeability [63]. Our data point out that the lack of *IL-13* reduces bacterial translocation in Abcb4^−/−^ mice. This is in accordance with previous reports showing the need for tight regulation of intestinal Th2 cytokines such as IL-4, but also IL-13, to maintain the intestinal barrier.

In *Abcb4*^−/−^ mice, but not in *Abcb4^−/−^/IL-13*^−/−^ mice, we found in the fecal microbiota enriched OTUs of *Prevotella* and *Oscillibacter*, which are both associated with intestinal pathology. *Prevotella* is overrepresented in the duodenum of cirrhotic patients [64], while increased *Oscillibacter* was shown to enhance gut permeability in diet-induced obese mice [65]. In contrast, *Prevotella* and *Oscillibacter* are known producers of anti-inflammatory metabolites, promoting the differentiation of anti-inflammatory Treg/Tr1 cells in the gut [66]. Surprisingly, in *Abcb4^−/−^/IL-13*^−/−^ mice, the genera *Prevotella* and *Oscillobacter* were not altered but the taxa *Lactobacillus*, *Roseburia*, and *Alistipes* were overrepresented. Correspondingly, in rats with obstructive jaundice, *Lactobacillus* has been shown to support hepatic barrier function through maintenance of the structure and function of tight junctions [67]. Of note, while a lack of IL-13 altered the microbial composition, it remains enigmatic whether these changes are directly related to improved hepatic barrier function and integrity. Although the gut microbiome is known to influence the bile acid metabolism [68], bile acids may also influence the composition of the intestinal microbiota directly or indirectly via the activation of the innate immunity [69], demonstrating the crosstalk between bile acids and microbiota [70]. Thus, normalization of serum and fecal bile acid composition in *Abcb4^−/−^IL-13*^−/−^ mice might be a direct consequence of the altered gut microbiome. Alternatively, normalized bile excretion might have influenced the composition of the gut microbiome [71]. The most important data of this study were summarized as a graphical abstract (Figure S7).

## 5. Conclusions

In conclusion, the prolonged liver integrity and normalization of microbial homeostasis demonstrate the therapeutic potential of blocking IL-13. This is the first study showing that *IL-13*-depletion could be a therapeutic approach for the treatment of patients with bile duct disorders.

## Figures and Tables

**Table 1:** Selected terms from functional annotation clustering of genes more than two-fold regulated in *Abcb4*^−/−^ and *Abcb4*^−/−^/*IL-13*^−/−^ livers.

Term	Count	*p*-Value *	Gene Name
Secondary metabolites biosynthesis, transport, and catabolism	18 14	4.9 × 10^−9^ 1.4× 10^−7^	*Cyp17a1*, *Cyp2b13*, *Cyp2a22*, *Cyp2c55*, *Cyp2c67*, *Cyp2c68*, *Cyp2g1*, *Cyp2c37*, *Cyp26a1*, *Cyp26b1*, *Cyp3a11*, *Cyp3a16*, *Cyp3a41b*, *Cyp3a44*, *Cyp4a10*, *Cyp4a12b*, *Cyp4a12a*, *Cyp4a31*, *Cyp4f18*, *Cyp7a1*, *Cyp7b1*
Extracellular matrix	24 26	1.2× 10^−9^ 3.0× 10^−13^	*Adamtsl2*, *Fras1*, *Adamts2*, *Adamts4*, *Ctsg*, *Col1a1*, *Col1a2*, *Col3a1*, *Col5a3*, *Col6a2*, *Col6a3*, *Dmbt1*, *Dpt*, *Fbn1*, *Lgals3*, *Lpl*, *Lum*, *Loxl1*, *Mgp*, *Mmp12*, *Mmp13*, *Mmp2*, *Mmp3*, *Mmp7*, *Mfap4*, *Mfge8*, *Slpi*, *Serpine1*, *Thbs1*, *Timp1*, *Zg16*
Retinol metabolism	15 11	2.4× 10^−7^ 2.4× 10^−5^	*Ugt2b37*, *Cyp2b13*, *Cyp2c55*, *Cyp2c68*, *Cyp2c37*, *Cyp26a1*, *Cyp26b1*, *Cyp3a11*, *Cyp3a16*, *Cyp3a41b*, *Cyp3a44*, *Cyp4a10*, *Cyp4a12b*, *Cyp4a12a*, *Cyp4a31*, *Dhrs9*
PPAR signaling pathway	13 8	3.0× 10^−6^ 1.6× 10^−3^	*Adipog*, *Cyp4a10*, *Cyp4a12b*, *Cyp4a12a*, *Cyp4a31*, *Cyp7a1*, *Fabp3*, *Fabp5*, *Lpl*, *Plin1*, *Ppard*, *Scd1*, *Scd3*, *Ucp1*
Muscle contraction	9 9	1.2× 10^−5^ 2.4× 10^−6^	*Cacna1s*, *Lmod3*, *Myom2*, *Mybpc1*, *Myh1*, *Myh2*, *Myh4*, *Myh7*, *Myl1*, *Tmod4*
Cell adhesion	30 23	3.5× 10^−5^ 2.1× 10^−4^	*Cd24a*, *Frem1*, *Ajap1*, *Cdh1*, *Clstn2*, *Clstn3*, *Cgref1*, *Cx3cl1*, *Col6a2*, *Ctgf*, *Cxadr*, *Dpt*, *Emb*, *Efs*, *Flrt1*, *Flrt3*, *Fbln7*, *Fblim1*, *Itga11*, *Itga8*, *Itgax*, *Lamc3*, *Lamc2*, *Mfap4*, *Mfge8*, *Myh10*, *Ncam1*, *Spp1*, *Siglecf*, *Sdk1*, *Svep1*, *Thbs1*, *Tinag*, *Tnfrsf12a*, *Ttyh1*, *Vcam1*
Steroid hormone biosynthesis	12 10	4.1× 10^−5^ 9.3× 10^−5^	*Ugt2b37*, *Cyp17a1*, *Cyp2b13*, *Cyp2c55*, *Cyp2c68*, *Cyp2c37*, *Cyp3a11*, *Cyp3a16*, *Cyp3a41b*, *Cyp3a44*, *Cyp7a1*, *Cyp7b1*, *Hsd3b1*, *Hsd3b5*, *Sult1e1*
Focal adhesion	18 14	3.2× 10^−4^ 4.7× 10^−4^	*Shc2*, *Actg1*, *Cav3*, *Col1a1*, *Col1a2*, *Col3a1*, *Col5a3*, *Col6a2*, *Col6a3*, *Ccnd1*, *Itga11*, *Itga8*, *Jun*, *Lamc3*, *Lamc2*, *Myl2*, *Pak6*, *Pdgfa*, *Spp1*, *Thbs1*
ECM receptor interaction	10 10	1.0× 10^−3^ 1.0× 10^−4^	*Col1a1*, *Col1a2*, *Col3a1*, *Col5a3*, *Col6a2*, *Col6a3*, *Itga11*, *Itga8*, *Lamc3*, *Lamc2*, *Spp1*, *Thbs1*
Chemical carcinogenesis	10 8	1.4× 10^−3^ 3.5× 10^−3^	*Ugt2b37*, *Cyp2b13*, *Cyp2c55*, *Cyp2c68*, *Cyp2c37*, *Cyp3a11*, *Cyp3a16*, *Cyp3a41b*, *Cyp3a44*, *Gstt3*, *Sult2a1*

* Upper *p-value*: *Abcb4*^−/−^ to wild type, bold: genes regulated in both cluster analyses, lower *p*-value: *IL-13*^−/−^ to *Abcb4*^−/−^/*IL-13*^−/−.^

## Supplementary Materials

The following are available online at https://www.mdpi.com/2073-4409/9/9/1949/s1, Figure S1: Serum alkaline phosphatase levels. Figure S2: Expression of genes responsible for BA synthesis and –transport, Figure S3: IL-13 knockout reduced ER stress, Figure S4: Epithelial proliferation and lymphocyte infiltration, Figure S5: Bacterial translocation to the liver was reduced in *Abcb4^−/−^/IL-13*^−/−^-mice. Figure S6: Graphical abstract.
