# Supplementary material for: IL-13 as Target to Reduce Cholestasis and Dysbiosis in Abcb4 Knockout Mice
Source: Cells. 2020 Aug 24;9(9):1949. doi: 10.3390/cells9091949 (PMC7564366; doi:10.3390/cells9091949)
Supplement: Supplementary file 1 [file cells-09-01949-s001.pdf]

cells MDPI

Suppl. Figure 1

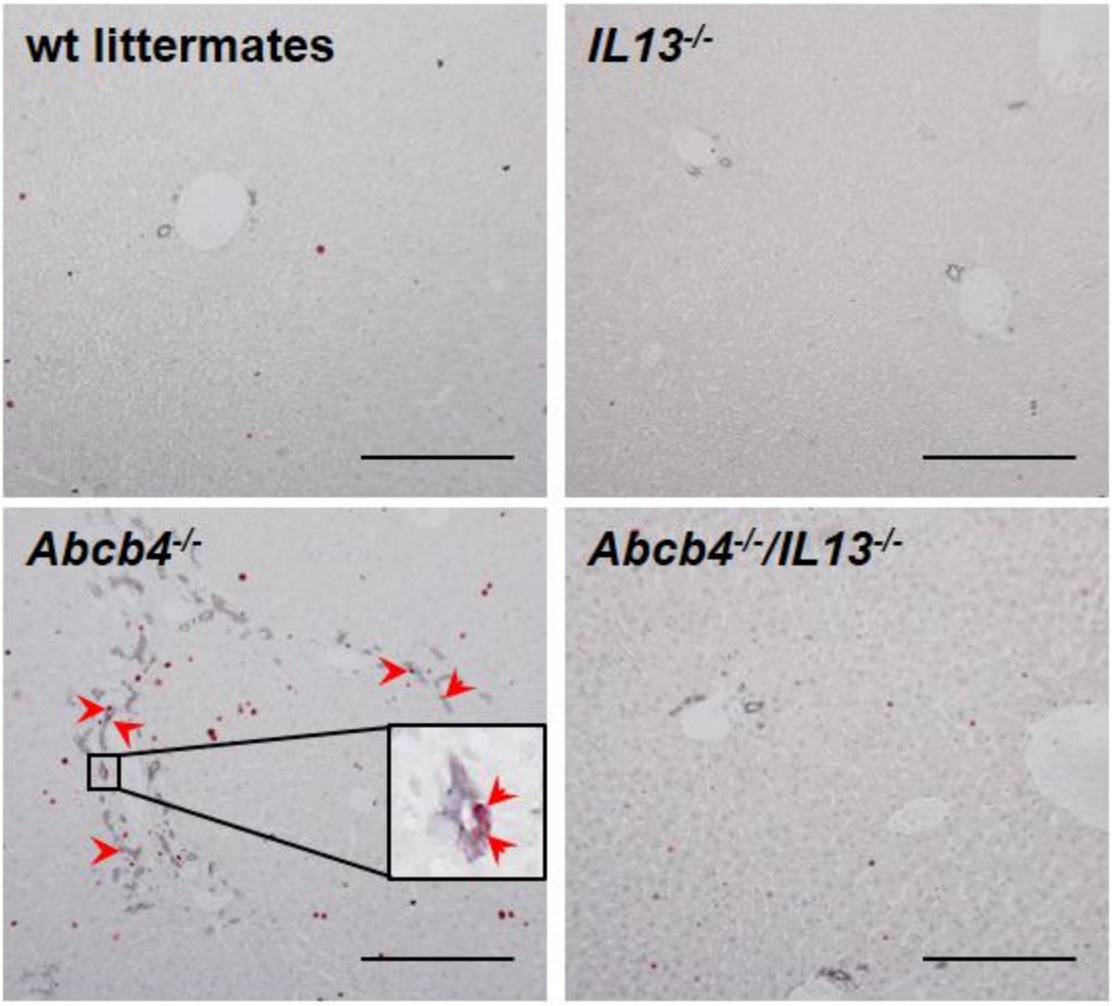

Figure 1. Ki67 staining (red) indicated proliferation of CK19$^+$-BEC (grey) in *Abcb4*$^{-/-}$ mice (arrowheads). Representative micrographs are shown. Bars 100 μm, magnification × 100.

.

Suppl. Figure 2

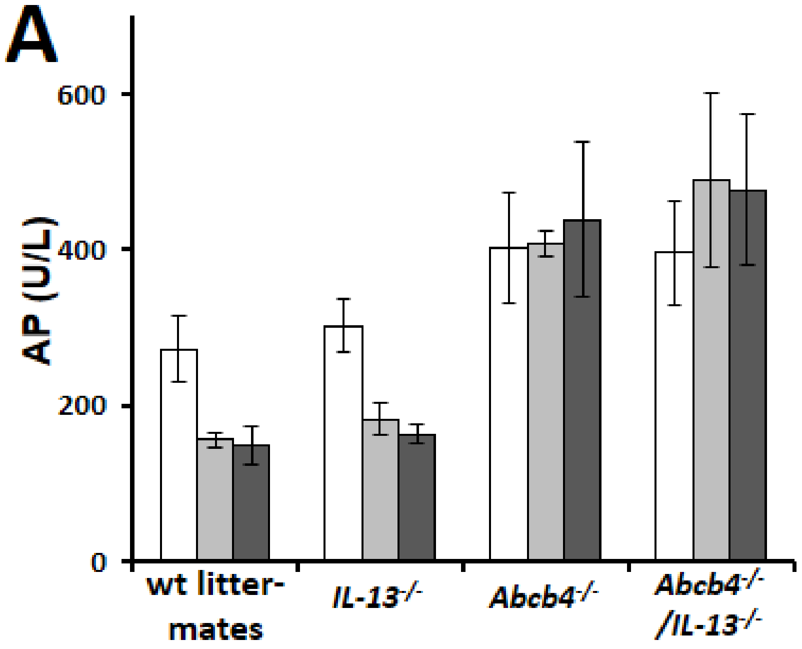

Figure 2. *IL-13* knockout did not alter serum alkaline phosphatase levels.

.

Suppl. Figure 3

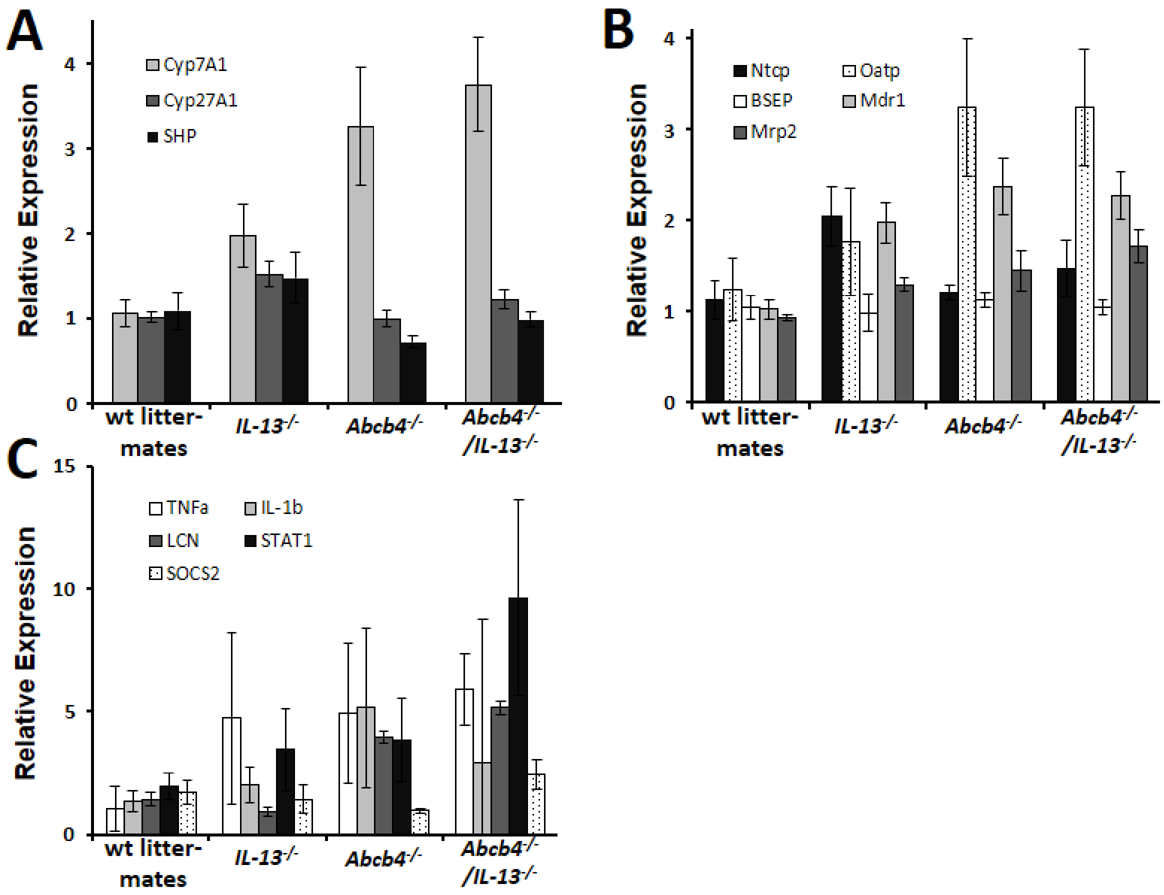

Figure 3. *IL-13* knockout did not alter BA synthesis, BA transporters, and hepatic inflammation. (**A**–**C**) Transcription of genes specific for bile acid synthesis (**A**), bile acid transporters (**B**), and inflammation (**C**) were not altered in 8 weeks old *Abcb4*$^{-/-}$*IL-13*$^{-/-}$-mice.

.

Suppl. Figure 4

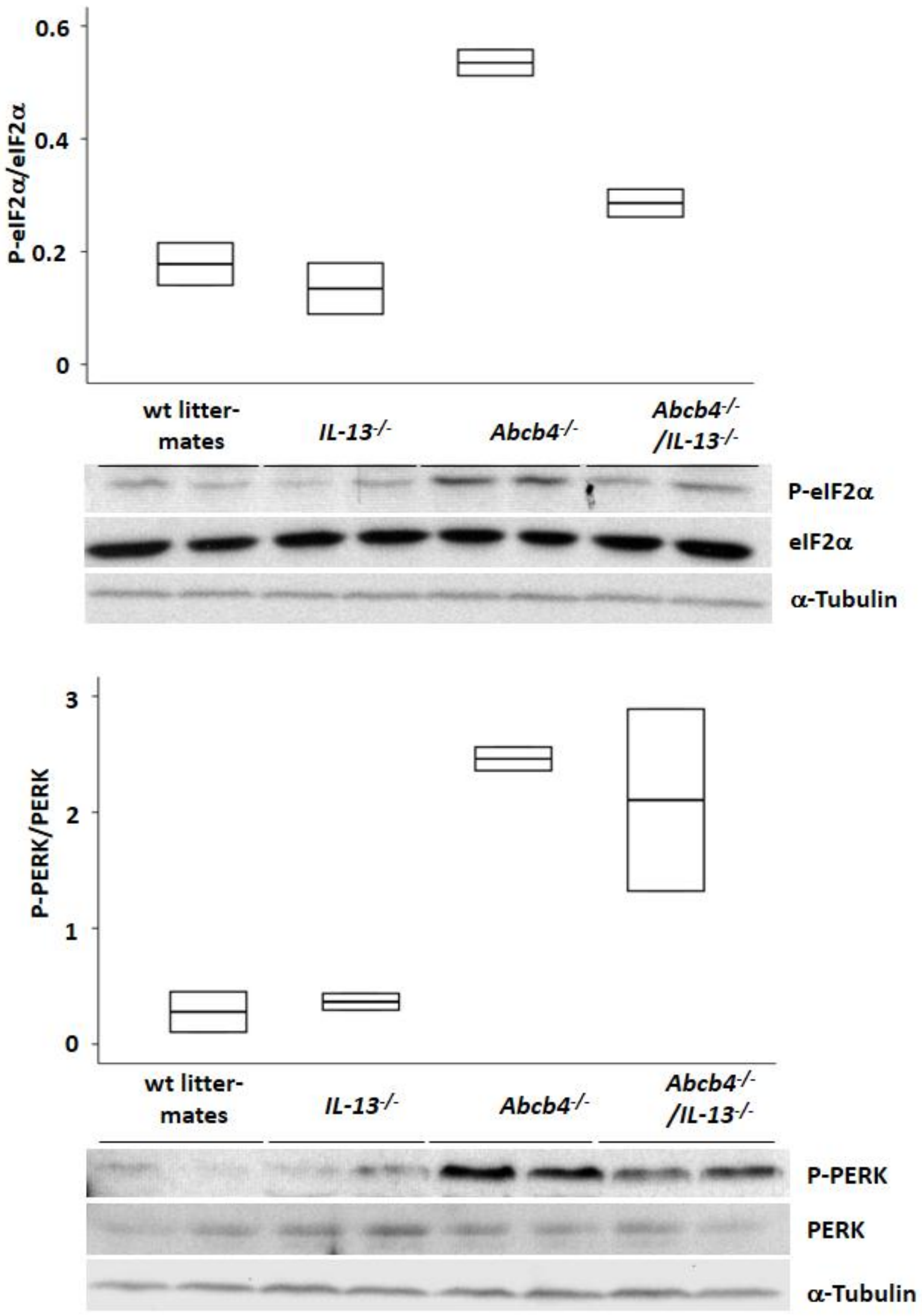

Figure 4. *IL-13* knockout reduced ER stress. Western blot demonstrated enhanced phosphorylation of PERK and eIF2α in *Abcb4*$^{-/-}$-mice and almost normalized phosphorylation in *Abcb4*$^{-/-}$*IL-13*$^{-/-}$-mice of 8 weeks. Equal protein loading was confirmed by detection of unphosphorylated proteins and α-Tubulin, respectively. A representative western blot is depicted.

.

Suppl. Figure 5

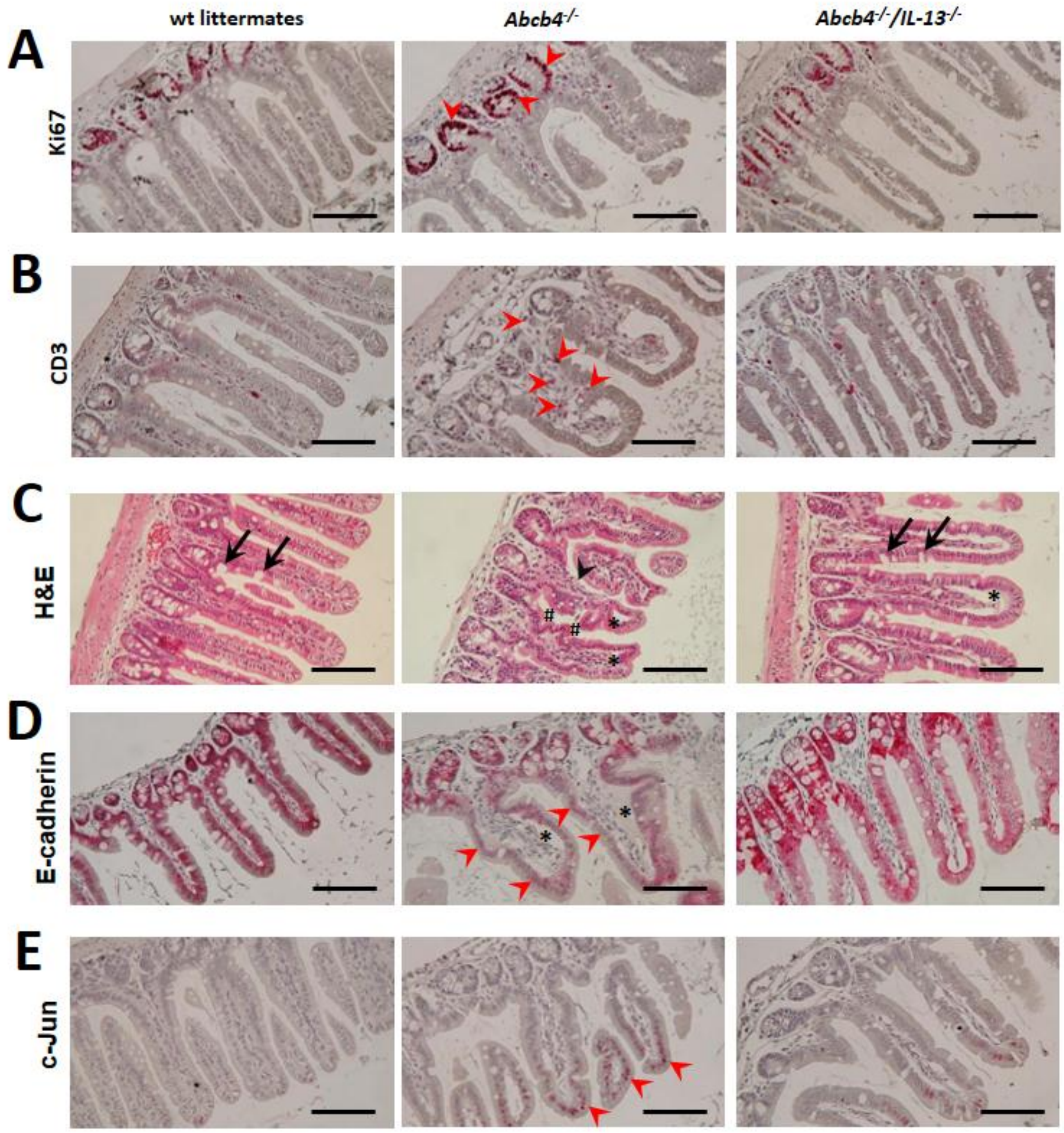

Figure 5. *IL-13* knockout reduced proliferation of ileal epithelium and infiltration of T-cells into ileal submucosa. (**A** and **B**) Immunohistochemical staining of Ki67 (**A**) and CD3 (**B**) revealed enhanced epithelial proliferation (red arrowheads) and infiltration of CD3$^+$-cells (red arrowheads) in *Abcb4*$^{-/-}$-mice and a normalized situation in *Abcb4*$^{-/-}$*IL-13*$^{-/-}$-mice. (**C**–**E**) An overview of the immunostained sections is shown in Figure 5E. Magnification 200 ×, bars 100 µm.

Suppl. Figure 6

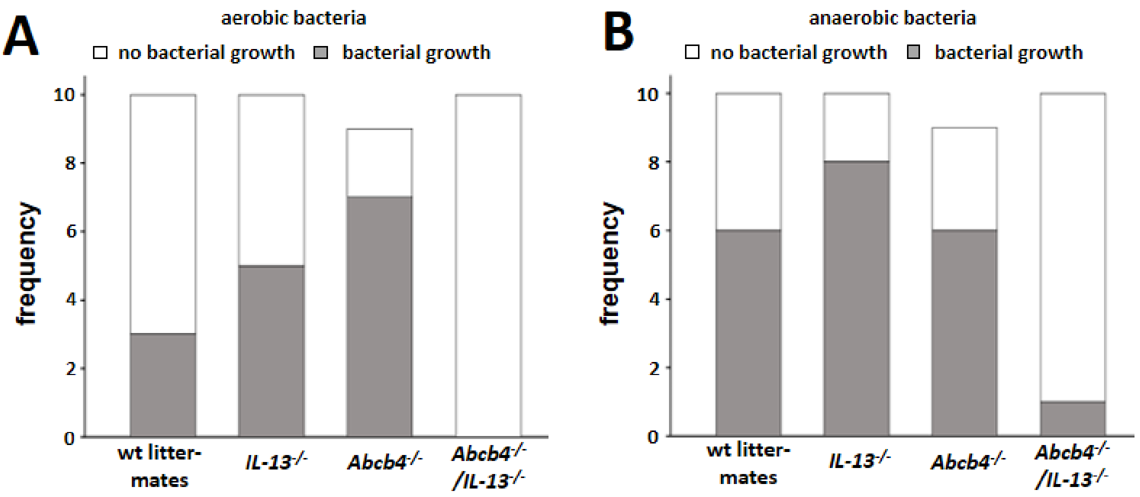

C

	Chi^2 (two-sided asymtotic significance)						
	all groups	wt-*IL-13*$^{-/-}$	wt-*Abcb4*$^{-/-}$	wt-*Abcb4*$^{-/-}$/*IL-13*$^{-/-}$	*IL-13*$^{-/-}$-*Abcb4*$^{-/-}$	*IL-13*$^{-/-}$-*IL-13*$^{-/-}$/*Abcb4*$^{-/-}$	*Abcb4*$^{-/-}$-*IL-13*$^{-/-}$/*Abcb4*$^{-/-}$
aerobic	0.005	0.36	0.037	0.060	0.21	0.010	<0.001
anaerobic	0.011	0.33	0.76	0.019	0.51	0.002	0.011

D

		No. of mice with bacterial translocation of aerobic species / anaerobic species			
**strain**		wt (n=10)	*IL-13*$^{-/-}$ (n=10)	*Abcb4*$^{-/-}$ (n=9)	*Abcb4*$^{-/-}$/*IL-13*$^{-/-}$ (n=10)
Lactobacillus spp.		2 / 4	3 / 3	5 / 5	-
Lactobacillus murinus		2 / 4	4 / 6	3 / 3	0 / 1
Staphylocoocus spp.		0 / 1	0 / 1	0 / 1	-
gram-positive cocci		-	0 / 1	-	-
alpha-hem. *Streptococcus*		-	1 / 0	4 / 0	-
Enterococcus spp.		-	-	1 / 0	-
Acinetobacter spp.		-	1 / 0	2 / 0	-
Bacteroides spp.		-	-	0 / 2	-
gram-labile bacilli		-	-	0 / 1	-

Figure 6. Bacterial translocation to the liver was reduced in *Abcb4*$^{-/-}$/*IL-13*$^{-/-}$-mice. Incidence of aerobic (**A**) and anaerobic (**B**) bacterial translocation to the liver. Data are reported as no. of animals with negative cultures (white bars) and positive cultures (grey bars) per total animals of each group. (**C**) The significance of differences in frequencies of bacterial translocation to the liver among the groups was calculated by Pearson´s chi-square test. (**D**) The table shows the no. of individual aerobic and anaerobic bacterial species that were identified in each group. Please note that some livers were positive for multiple species.

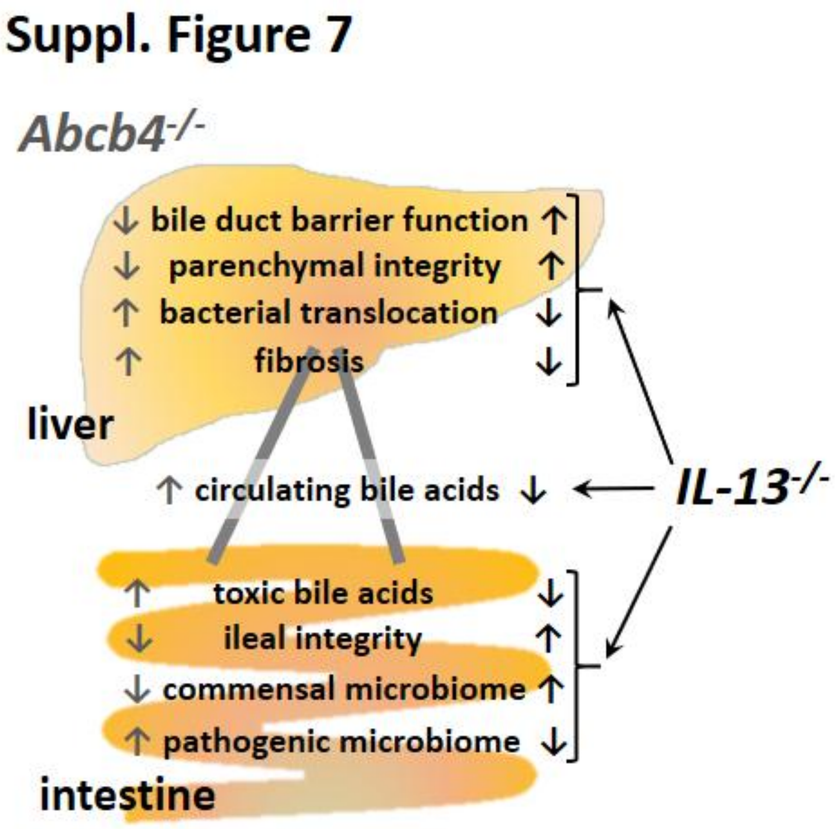

Figure 7. The major findings of this study were summarized as a graphical abstract. .
